# Revealing the closed pore formation of waste wood-derived hard carbon for advanced sodium-ion battery

**DOI:** 10.1038/s41467-023-39637-5

**Published:** 2023-09-27

**Authors:** Zheng Tang, Rui Zhang, Haiyan Wang, Siyu Zhou, Zhiyi Pan, Yuancheng Huang, Dan Sun, Yougen Tang, Xiaobo Ji, Khalil Amine, Minhua Shao

**Affiliations:** 1 Hunan Provincial Key Laboratory of Chemical Power Sources, College of Chemistry and Chemical Engineering, Central South University, Changsha, 410083 P.R. China; 2 Department of Chemical and Biological Engineering, Energy Institute, Hong Kong University of Science and Technology, Clear Water Bay, Kowloon Hong Kong, P.R. China; 3 Collaborative Innovation Center of Sustainable Energy Materials, Guangxi Key Laboratory of Electrochemical Energy Materials, Guangxi University, Nanning, 530004 P.R. China; 4 Chemical Sciences and Engineering Division, Argonne National Laboratory, Lemont, IL 60439 USA

**Keywords:** Batteries, Batteries, Batteries

## Abstract

Although the closed pore structure plays a key role in contributing low-voltage plateau capacity of hard carbon anode for sodium-ion batteries, the formation mechanism of closed pores is still under debate. Here, we employ waste wood-derived hard carbon as a template to systematically establish the formation mechanisms of closed pores and their effect on sodium storage performance. We find that the high crystallinity cellulose in nature wood decomposes to long-range carbon layers as the wall of closed pore, and the amorphous component can hinder the graphitization of carbon layer and induce the crispation of long-range carbon layers. The optimized sample demonstrates a high reversible capacity of 430 mAh g^−1^ at 20 mA g^−1^ (plateau capacity of 293 mAh g^−1^ for the second cycle), as well as good rate and stable cycling performances (85.4% after 400 cycles at 500 mA g^−1^). Deep insights into the closed pore formation will greatly forward the rational design of hard carbon anode with high capacity.

## Abstract (web summary)

It is essential to investigate the formation mechanism of closed pore, which contributes to low-voltage plateau capacity of hard carbon anode in sodium ion batteries. Herein, the authors explore the impact of wood precursor components and carbonization temperature on closed pore formation in hard carbon for enhanced battery performance.

## Introduction

Sodium-ion batteries (SIBs) are one of the most promising candidates of lithium-ion batteries (LIBs) for large-scale electrical energy storage and low-speed electric vehicles due to the low cost and abundance of sodium resources^1^. Although plenty of cathode materials have been developed, the lack of high-performance anode materials greatly impedes the further improvement of energy density in SIBs. For instance, graphite, which is a benchmark anode material for LIBs, demonstrates limited sodium storage capacity because of the instability of sodium-graphite intercalation compounds^2^. In this respect, the innovation of affordable and achievable anode materials with remarkable performance is of great significance.

Among the various reported anode materials, hard carbon is the most promising one for practical SIBs owing to its balanced performances in terms of moderate specific capacity (~300 mAh g^−1^), low operating potential (~0.2 V), low cost, and long cycle life^3^. Noting that the sodium storage mechanism of hard carbon is still controversial, which severely hinders the further improvement of specific capacity and rate capability^2,4^. As is well known, hard carbon is composed of randomly oriented, curved, and defective graphene nanosheets, turbostratic structure with large interlayer distance^5–7^. Recent declaration on the sodium storage mechanism by Hu et al. pointed out that the sloping and plateau capacity relates to the complex turbostratic structure and internal closed pore, respectively^8^. It should be noted that the exact nature of the sodium stored within the pores is disputed, with some observing metallic sodium, whilst only ionic sodium is present in other systems^8–11^. The low-voltage plateau capacity is the main contributor to the higher energy density of hard carbon anodes for SIBs^3,12,13^. Therefore, it is urgent to gain a deep understanding of the sodium storage mechanism and elaborate on how to design the microstructure of hard carbon.

According to previous reports, a low-cost method to prepare hard carbon is carbonizing biomass, such as apricot shells^14^, rice husks^15^, lotus seedpods and stems^16,17^, banana peels^18^, coconut oil^19^, palm fruit calyx^20^, and cotton^7^. As well known, large amounts of waste wood are used to generate electricity by burning, discarded to rubbish, and plant agriculture^21^. Fabrication of higher-value products from waste wood is an important route to improve its economic competitiveness and utilization efficiency^21^. Moreover, wood-derived materials show unique advantages in terms of resource abundance, renewability, sustainability, and material cost, which are intriguing for electrochemical energy storage, especially for the low-cost stationary grid and portable electronics^22^. Hence, these unique advantages and significance inspire researchers to develop high-performance hard carbon materials derived from waste wood. Wang et al. proposed a pore-forming-opening strategy to achieve a high-capacity hard carbon anode with the precursor of natural balsa (439 mAh g^−1^ at 100 mA g^−1^)^23^. Our group also successfully regulated the pore structure of rose wood-derived hard carbon via chemical pre-treatment and low-temperature pyrolysis^24^. The as-prepared carbon anode delivered a capacity of 326 mAh g^−1^ at 20 mA g^−1^. Nevertheless, a more systematic formation mechanism of closed pores has not been established sufficiently in wood-derived carbon materials.

In this work, we investigate the effect of different components (crystalline cellulose and amorphous hemicellulose/lignin) in natural wood precursor and carbonization temperature on the formation of closed pore structure in the derived hard carbon. With the support of in-situ or ex-situ characterization techniques, we reveal that long graphite-like layers originated from the decomposition of crystalline cellulose serve as the wall of closed pore structure, while the amorphous hemicellulose and lignin are the inhibitors that prevent the over-graphitization of carbon layer during the high-temperature carbonization. Besides, the length of graphite-like carbon layer increases with the increase in carbonization temperature, accelerating the formation of closed pore structure. Meanwhile, the optimum hard carbon derived from waste wood displays good rate capability, high reversible plateau capacity, and stable cycling performance. The proposed closed pore formation mechanism for waste wood-derived carbon can motivate us to develop hard carbon anodes with high plateau-region capacity towards high-energy density SIBs.

## Results

### The role of crystalline cellulose content on forming closed pore in hard carbon

**Fig. 1:**
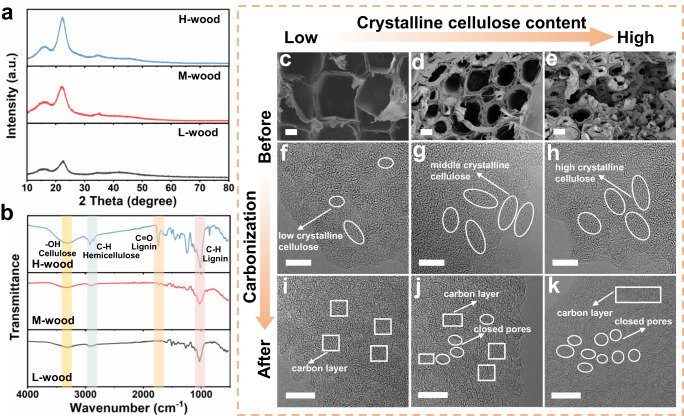
Physico-chemical characterization of different wood precursors before and after carbonization. **a** XRD patterns and (**b**) FTIR spectra of L-wood, M-wood and H-wood samples. SEM images of (**c**–**e**) L-wood, M-wood and H-wood samples respectively and (**f**–**h**) the corresponding HRTEM images. HRTEM images of (**i**–**k**) L-1500, M-1500 and H-1500 samples respectively. Scale bars: 10 μm (**c**–**e**); 5 nm (**f**–**h**); 10 nm (**i**–**k**).

As is well known, wood is a complex composite composed of cellulose, hemicellulose, lignin, and so on^25–28^. To better uncover the correlation between the composition of wood precursor and the microstructure in its derived hard carbon, three wood precursors with low, middle and high contents of crystalline cellulose were chosen and noted as L-wood, M-wood and H-wood, respectively. To quantify the cellulose crystallinity in wood precursors, powder X-ray diffraction (XRD) tests were performed. As shown in Fig. 1a, three peaks at 17°, 22°, and 35° are indexed to the crystalline cellulose^29^. According to the intensity of the characteristic peak at 22° and the Segal method^30^, the crystalline cellulose contents for L-wood, M-wood and H-wood are calculated to be 49.9%, 53.2% and 68.4%, respectively. The crystallinity index values of cellulose in L-wood, M-wood and H-wood are 0.97, 0.98 and 1.09, respectively, which are calculated based on Nelson and O’connor method according to the Fourier transforms infrared (FTIR) spectra (Fig. 1b)^31^. The morphology and microstructure of wood precursors were investigated with SEM and HRTEM. L-wood (Fig. 1c and Supplementary Fig. 1a, b) mainly consists of many uniform tracheid cells, similar to a honeycomb-like structure. As the wood cellulose crystallinity increases, both M-wood (Fig. 1d and Supplementary Fig. 1c, d) and H-wood (Fig. 1e and Supplementary Fig. 1e, f) show more complicated micromorphology with thicker walls and more fibers. HRTEM images demonstrate that L-wood (Fig. 1f) is composed of disordered domains and some regions of short-range order (marked by white cycle). More long-range order structures are observed in M-wood (Fig. 1g) and H-wood (Fig. 1h) precursors, which are ascribed to the higher crystalline cellulose content.

After heat treatment, all wood precursors were well carbonized and decreased in size (Supplementary Fig. 2). SEM images show that these as-prepared hard carbon samples still retain natural pores and well-connected structure (Supplementary Fig. 3). To investigate the microstructure of hard carbon samples formed at 1500 °C, HRTEM images are shown in Fig. 1i–k. Figure 1i demonstrates the highly disordered nature of L-1500 sample prepared from carbonized L-wood precursor, and it is difficult to identify obvious closed pore areas and long graphite-like layers. Nevertheless, some closed pores and long graphite-like layers can be clearly observed in M-1500 (Fig. 1j) and H-1500 (Fig. 1k). Remarkably, H-1500 sample possesses abundant graphite-like layers longer than 5 nm and closed pores larger than 2 nm, where they stack into turbostratic closed void domains (marked by the white cycles). The rich closed pores in H-1500 sample might be related to the high crystalline cellulose content of its wood precursor, which can be decomposed to the long graphite-like layers during the carbonization process to surround and shrink the sites.

**Fig. 2:**
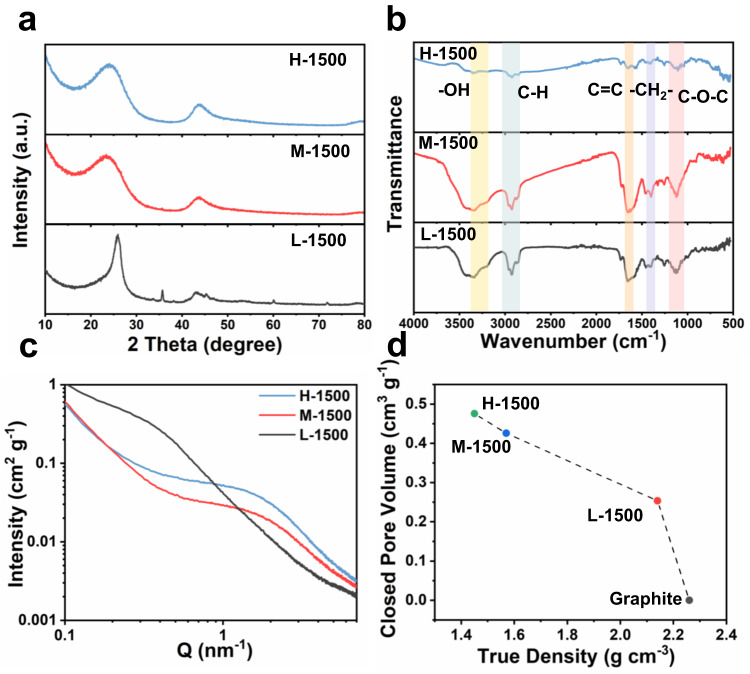
The physicochemical characterization of L-1500, M-1500 and H-1500. **a** XRD patterns, (**b**) FTIR spectra, (**c**) SAXS patterns, and (**d**) the relationship between the closed pore volume and the true density.

The physicochemical properties of L-1500, M-1500 and H-1500 were further characterized. XRD patterns of H-1500 and M-1500 have two peaks at about 23° and 43°, corresponding to the (002) and (100) crystal planes of disordered graphite domains, respectively, which are typical characteristics of hard carbon (Fig. 2a)^3^. In contrast, L-1500 exhibits a sharp peak at 25.8°, demonstrating that the graphite-like layers are highly stacked and the corresponding interlayer spacing is close to that of the graphite. FTIR spectroscopy analysis was conducted to interpret the degree of cellulose decomposition in different wood-derived carbon. As shown in Fig. 2b, FTIR spectra verify the existence of -OH (3338 cm^−1^), −CH_2_− (1465 cm^−1^) and C−O−C (1125 and 1250 cm^−1^) in all carbon samples^22^. Noting that, the absorption peak intensities of −OH, −CH_2_−, −CH−, and C−O−C in H-1500 significantly decrease in comparison to those of H-wood, indicating that the H-wood precursor underwent more sufficient carbonization and had an underlying effect on the internal structure formation. X-ray photoelectron spectra (XPS) demonstrate that no other heteroatom is found except a small number of O atoms (~5 at%) in hard carbon samples (Supplementary Fig. 4a and Supplementary Table 1). The peaks at 284.77, 285.38, 285.9 and 288.9 eV in the C 1s high-resolution spectra are ascribed to *sp*^2^, *sp*^3^, C-O and O = C-O, respectively (Supplementary Fig. 4b)^6,15,21^. The Raman spectra of hard carbons generally exhibit broad peaks around 1345 cm^−1^ (D-band) and 1586 cm^-1^ (G- band). The integrated intensity ratio of G-band and D-band, I_G_/I_D_, can reflect the defects concentration along with the graphene sheets^1,5^. As seen in Supplementary Fig. 5, the I_G_/I_D_ value of wood-derived carbon is gradually increased (1.15 to L-1500, 1.17 to M-1500, 1.295 to H-1500) indicating that H-1500 processes higher disordered degree due to the formation of more closed pores^12^. N_2_ and CO_2_ physisorption tests (Supplementary Fig. 6 and Supplementary Table 2) were performed to reveal the pore structure in hard carbon samples. Compared with L-1500 and M-1500, H-1500 possesses the smallest specific surface area (SSA) and the lowest content of micropores and mesopores.

As known, N_2_ and CO_2_ adsorption/desorption measurement only can probe the open surface porosity and is not sensitive to the internal closed porosity. To better describe the properties of closed pores in hard carbon samples, the small-angle X-ray scattering (SAXS) test was conducted. SAXS patterns reveal broad humps at the scattering vector *Q* of 1–2 nm^−1^, which can be calculated by the following equation^3,8,13,32,33^:

1 $$Q=\frac{4\pi {{\sin }}\theta }{\lambda }$$

where λ = 1.541 Å is the X-ray wavelength and θ is half the scattering angle. The humps are attributed to closed pores in the carbon matrix, including micrometer-sized and nanometer-sized voids between *sp*^2^ graphite layers^8,32^. According to SAXS patterns of hard carbon, H-1500 possesses an obvious peak around 1 nm^−1^, indicating the radius increase of closed pores and high content of closed pores (Fig. 2c)^8^. The true density analysis is also an effective technique to characterize the closed pore. During the true density test, the volume of open pores and interparticle space can be well excluded. Therefore, the true density analysis can measure the total volume of closed pores and the solid portion. As a reference, the ideal graphite anode, regarded as a perfect crystal layered material without closed pores, possesses a high true density value of 2.26 g cm^−3^ (Fig. 2d)^12^. Nevertheless, as the content of crystalline cellulose increases in wood precursors, the true density of the corresponding hard carbon decreases, which reveals the increased closed pore volume. For comparison, H-1500 carbonized from H-wood with the most crystalline cellulose owns the highest closed pore volume of 0.48 cm^3^ g^−1^. Obviously, the closed pore content in hard carbon is related to the crystalline cellulose content in its wood precursor^8^.

### The role of amorphous composition on forming closed pore in hard carbon

**Fig. 3:**
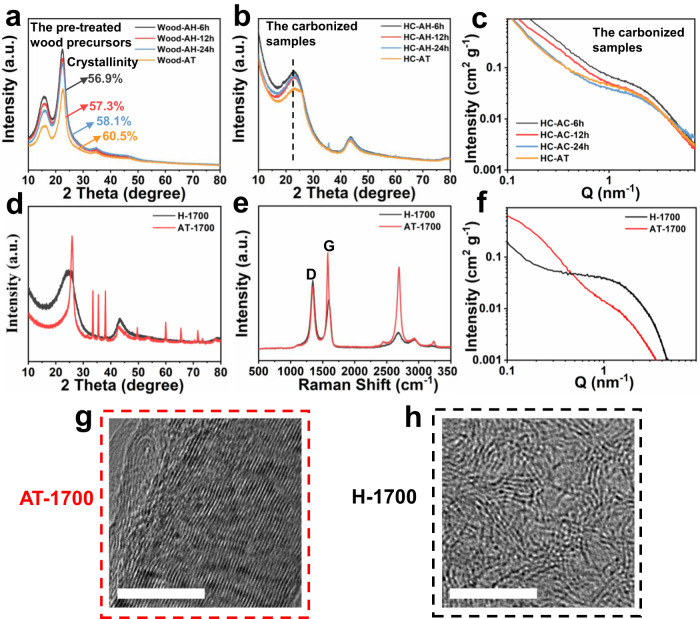
Physico-chemical characterization of the pretreated precursors and the derived hard carbon samples. XRD patterns of (**a**) wood precursors pretreated with acid or alkali and (**b**) the corresponding carbonized samples at 1500 °C. **c** SAXS patterns of the carbonized samples at 1500 °C. The material characterization of H-1700 and AT-1700. **d** XRD patterns, (**e**) Raman spectra and (**f**) SAXS patterns (**f**) of H-1700 and AT-1700; TEM images of (**g**, **h**) AT-1700 and H-1700. Scale bars: 10 nm (**g**, **h**).

To reveal the relationship between the amorphous components (lignin and hemicellulose) on the closed pore formation, acid and subsequent alkali hydrolysis treatment was employed to remove the amorphous composition in H-wood (see more experimental details in Experimental Section)^34,35^. The FTIR spectra of wood precursors pretreated with acid (Wood-AH-6h, Wood-AH-12h and Wood-AH-24h) or alkali (Wood-AT) show that the peak intensity of characteristic functional groups related to hemicellulose and lignin^12^ weakens with the extended processing time, revealing that hemicellulose and lignin could be effectively removed, while the crystalline cellulose was maintained (Supplementary Fig. 7). This conclusion can be further verified by the increased cellulose crystallinity along with the removal of hemicellulose and lignin (Fig. 3a). These pretreated wood precursors were carbonized at 1500 °C for further investigation. The XRD patterns of as-prepared hard carbon samples only show two broad diffraction peaks ((002) and (100) crystal planes) similar to that of H-1500 without pretreatment, which is attributed to their typical disordered carbon structure (Fig. 3b). Compared with HC-AH-6h, HC-AH-12h and HC-AH-24h, the (002) peak of HC-AT shifts to a higher degree, which means narrower carbon interlayer distance and more obvious graphitization tendency. According to the SAXS patterns of hard carbon after treatment, the peaks around 1–2 nm^−1^ decrease with the increased treatment time, meaning that the formed closed pores obviously decrease after removing the amorphous content (Fig. 3c). Obviously, the content of amorphous composition in wood precursor has an important influence on the pore structure and the graphitization degree of the derived hard carbon.

As is known to all, high pyrolysis temperature can provide a strong driving force to elevate the graphitization degree and closed pore content of hard carbon^12^. To better highlight the function of amorphous composition on tuning the microstructure of hard carbon, the pristine H-wood and the treated wood-AT were carbonized at 1700 °C to prepare H-1700 and AT-1700, respectively. Even at a pyrolysis temperature up to 1700 °C, the XRD pattern of H-1700 still maintains a typical feature of highly disordered carbon (Fig. 3d). In contrast, AT-1700 exhibits a sharp peak at 26°, which is characteristic of highly graphitic carbon structure formed at high temperature, further confirming that amorphous region is a barrier for long-range graphitization of carbon layer. Raman spectra also prove the variation of graphitization degree, and the intensity ratio (I_D_/I_G_) of hard carbon samples decreases from 1.384 (H-1700) to 0.646 (AT-1700) after removing the amorphous composition (Fig. 3e). According to the SAXS patterns in Fig. 3f, the H-1700 with amorphous region retaining induces a more obvious peak at 1-2 nm^-1^, indicating the H-1700 possesses more closed pores. The visualized differences between H-1700 and AT-1700 on microstructures are shown in HRTEM images (Fig. 3g, h). AT-1700 exhibits a highly ordered and parallel graphitic-like layers structure in Fig. 3g, while some closed pores still can be observed in H-1700 in Fig. 3h, which should be induced by the folding of long-range and curved graphite layers. Hence, the amorphous regions not only effectively prevent the graphitization of wood-derived carbon, but also facilitate the formation of closed pore structures.

### The influence of temperature on forming closed pore of hard carbon

**Fig. 4:**
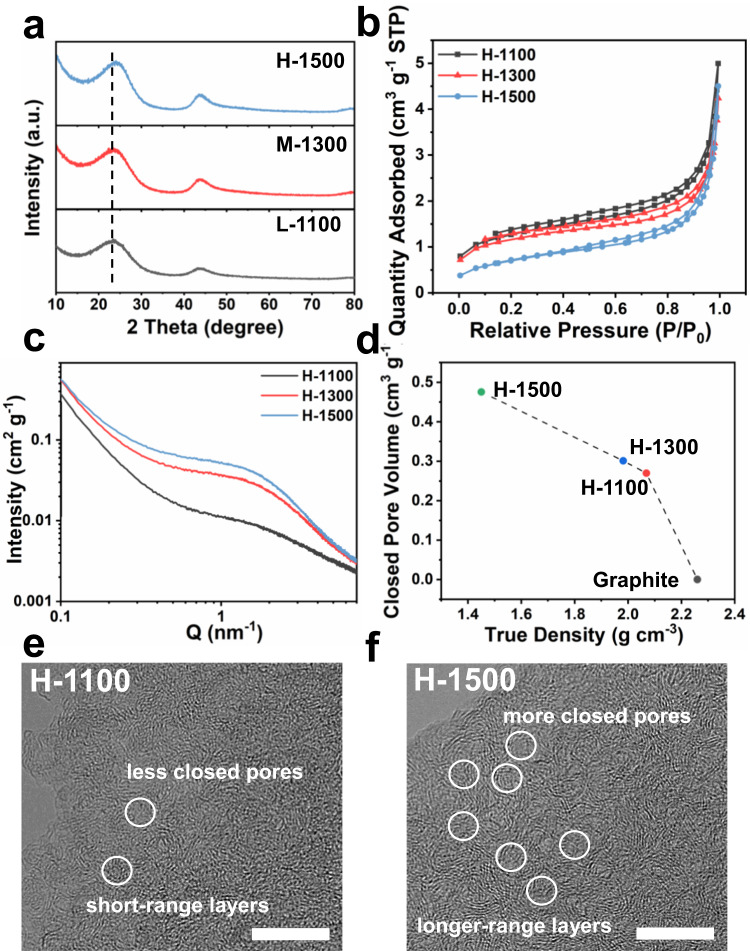
Physico-chemical characterization of hard carbon samples at different carbonization temperatures. **a** XRD patterns, (**b**) N_2_ adsorption-desorption isotherms, (**c**) SAXS patterns and (**d**) the relationship between the closed pore volume and true density of H-1100, H-1300 and H-1500. TEM images of (**e**) H-1100 and (**f**) H-1500. Scale bars: 10 nm (**e**, **f**).

The influence of carbonization temperature on the microstructure of hard carbon was also investigated. As displayed in Supplementary Fig. 8, all hard carbon samples prepared at different pyrolysis temperatures (H-1100, H-1300 and H-1500) show similar honeycomb-like structure with clear micro/nano channels and pores. As the pyrolysis temperature increases, the content of the residual oxygen atom is slightly reduced according to the XPS result (Supplementary Fig. 9 and Supplementary Table 3). In Fig. 4a, H-1100, H-1300 and H-1500 show similar XRD patterns, except that the (002) diffraction peak for H-1500 shifts to a larger angle owing to the decreased interlayer distance. As an almost universal cognizance, higher carbonization temperature means fewer defects and higher graphitization degree, resulting in a lower intensity ratio of I_D_/I_G_. Nevertheless, the value of I_D_/I_G_ increases from 1.15 to 1.295 with the elevated pyrolysis temperature (Supplementary Fig. 10), which might be ascribed to abundant closed pores in H-1500 and they influence the degree of disorder value. Moreover, the SSA and the pore volume of H-wood derived hard carbon samples decrease with the increase in heat temperature (Fig. 4b, Supplementary Fig. 11, 12 and Supplementary Table 4). These results of SAXS patterns and true density tests in Fig. 4c, d show that on increasing carbonization temperature, the formation of closed pores between graphene basal planes keeps growing. This is because the high crystallinity cellulose is fused to form a graphite-like layer, which shrunk to form closed pores^29^. The closed pore structure for H-1500 is further confirmed by TEM. The TEM images show that the orientation of the graphite-like domains becomes clearer and short graphite-like layers grow into long-range layers with increasing pyrolysis temperature (Fig. 4e, f and Supplementary Fig. 13). The high pyrolysis temperature facilitates the shift and fold of the graphite-like layers, generating more closed pore surrounded by several parallel carbon layers for sodium storage.

### The carbonization model of wood

**Fig. 5:**
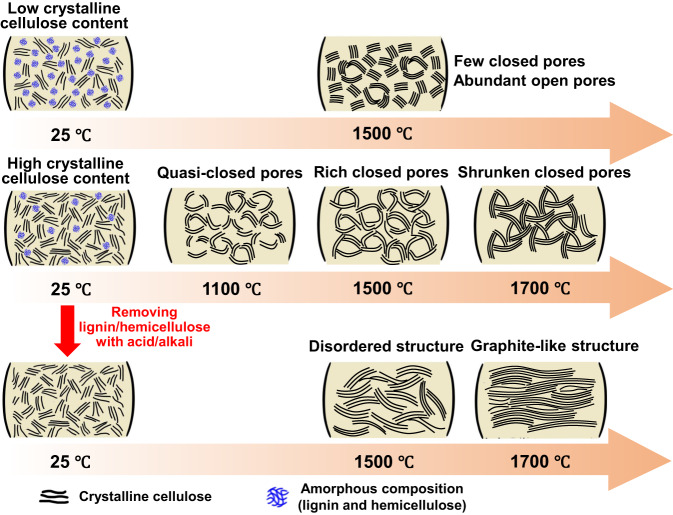
A formation mechanism of closed pores based on the observation in this work. Both the composition of wood precursor (crystallinity cellulose and amorphous hemicellulose/lignin) and the carbonization temperature play important roles in affecting the microstructure (such as number, size and wall thickness) of closed pores.

Based on the above-mentioned characterization results, it is clear that the formation of closed pore structure involves two key factors. The first one is enough length of graphene sheets in hard carbon. The second is that the produced graphene sheets can be effectively bent and disordered. The roles of wood components and calcination temperature in the formation of closed pores are first proposed. As illustrated in Fig. 5, the crystalline cellulose content is crucial to achieving closed pore structure during the pyrolysis process. The crystallinity cellulose can decompose and is carbonized to generate long graphene sheets as the walls of the closed pore, which will shrink to form closed pore structure. Meanwhile, the amorphous component is not only an active site to form closed pores but also a barrier to prevent wood-derived carbon from graphitization tendency. The wood precursor with low crystallinity cellulose shows few closed pores and abundant open pores (micropores and mesopores) even at a high annealing temperature of 1500 °C. Moreover, graphene sheets are easy to stack and result in the thick wall of close pore owing to the obstruction of these amorphous components. In contrast, more closed pores with thin wall appear after the wood precursor with high crystallinity cellulose is carbonized at the same temperature. The carbonization temperature also plays an important role in the formation of pore structure of wood-derived hard carbon. Some curved carbon layers also tend to fold and form “quasi-close pore” at the relatively low pyrolysis temperature. Although beneficial for the formation of new close pores, the high temperature also facilitates the migration, stacking and growth of graphitic layers, resulting in the shrunken closed pores.

### Electrochemical performance and storage mechanism of hard carbon

**Fig. 6:**
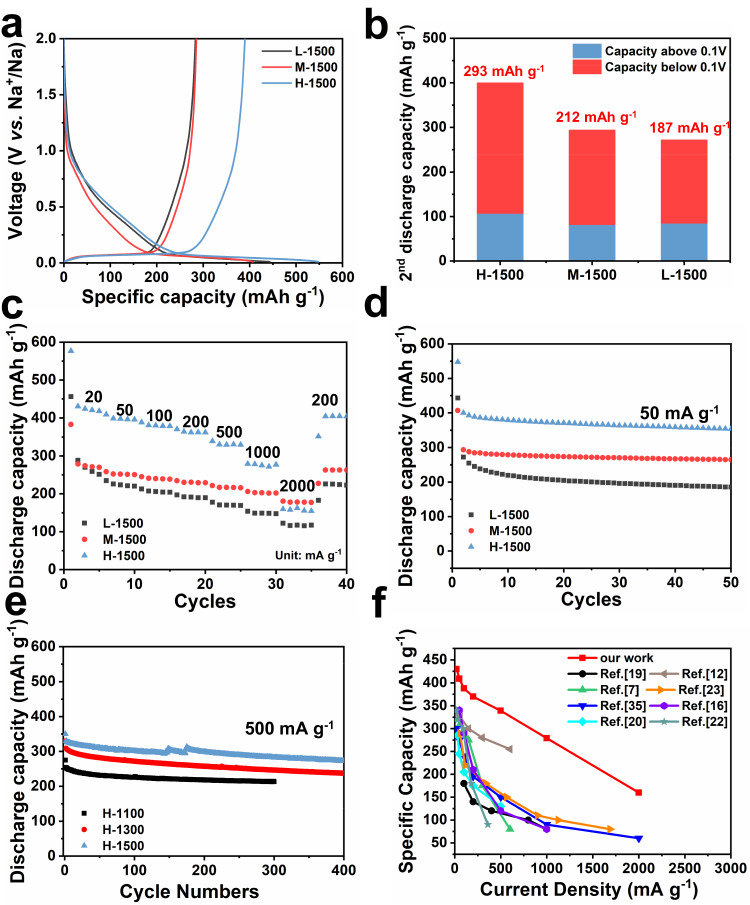
Battery performance. The (**a**) charge/discharge profiles at 50 mA g^−1^, (**b**) 2^nd^ discharge capacity contributed from slope and plateau region, (**c**) rate and (**d**) cycling performance of hard carbon samples prepared at 1500 °C. **e** The long-term cycling performance of hard carbon samples prepared at different temperatures. **f** Comparison of rate performance with the typical biomass-derived hard carbon reported previously for sodium storage.

The electrochemical performances of as-prepared hard carbon samples were first tested after assembling Na-ion half cells. The charge/discharge curves of L-1500, M-1500 and H-1500 can be divided into slope region (above 0.1 V) and plateau region (below 0.1 V), demonstrating typical Na ion storage behavior of hard carbon (Fig. 6a). Interestingly, H-1500 delivers a much higher initial reversible capacity of 390 mAh g^−1^ in comparison to L-1500 (284 mAh g^−1^) and M-1500 (286 mAh g^−1^) at 50 mA g^−1^. Figure 6b shows the plateau discharge capacities of three samples in the second cycle, and the corresponding value is 187, 212 and 293 mAh g^−1^, respectively. Such a high plateau capacity of H-1500 should be attributed to the abundant closed pores, which have been regarded as excellent Na^+^ storage sites in many references^3,8,13^. The similar phenomenon that the discharge capacity or plateau capacity varies with their closed pore content is also observed in other hard carbon electrodes (Supplementary Fig. 14). Such results reveal the correlation between Na^+^ storage capacity and closed pore structure. Also, H-1500 exhibits better rate capability with a high specific capacity of 280 mAh g^−1^ at 1000 mA g^−1^, while M-1500 and L-1500 possess a capacity of 202 and 147 mAh g^−1^, respectively (Fig. 6c). Nevertheless, when the current density further increases to 2000 mA g^−1^, the discharge capacity of H-1500 electrode is slightly lower than that of M-1500 electrode. Note that the discharge capacity of H-1500 electrode at 20 mA g^−1^ is as high as 430 mAh g^−1^. It is speculated that the sluggish kinetics of closed pore as Na^+^ storage site results in inferior rate performance. All three samples show excellent cycling stability (Fig. 6d). In particular, even at a current density of 500 mA g^−1^, H-1500 still maintains a capacity of 280 mAh g^−1^ after 400 cycles (Fig. 6e). When further increased the mass loading from 1.8 mg cm^−2^ to 3.7 mg cm^−2^, H-1500 electrode delivers an initial capacity of 273 mAh g^−1^ with a capacity retention of 89.9% at 1 A g^−1^ for 250 cycles (Supplementary Fig. 15). Compared with previous reports^7,12,16,19,20,22,24,36^ on biomass-derived carbon materials in common ether or ester electrolytes, the as-prepared H-1500 here demonstrates much higher reversible capacity and rate performance (Fig. 6f).

**Fig. 7:**
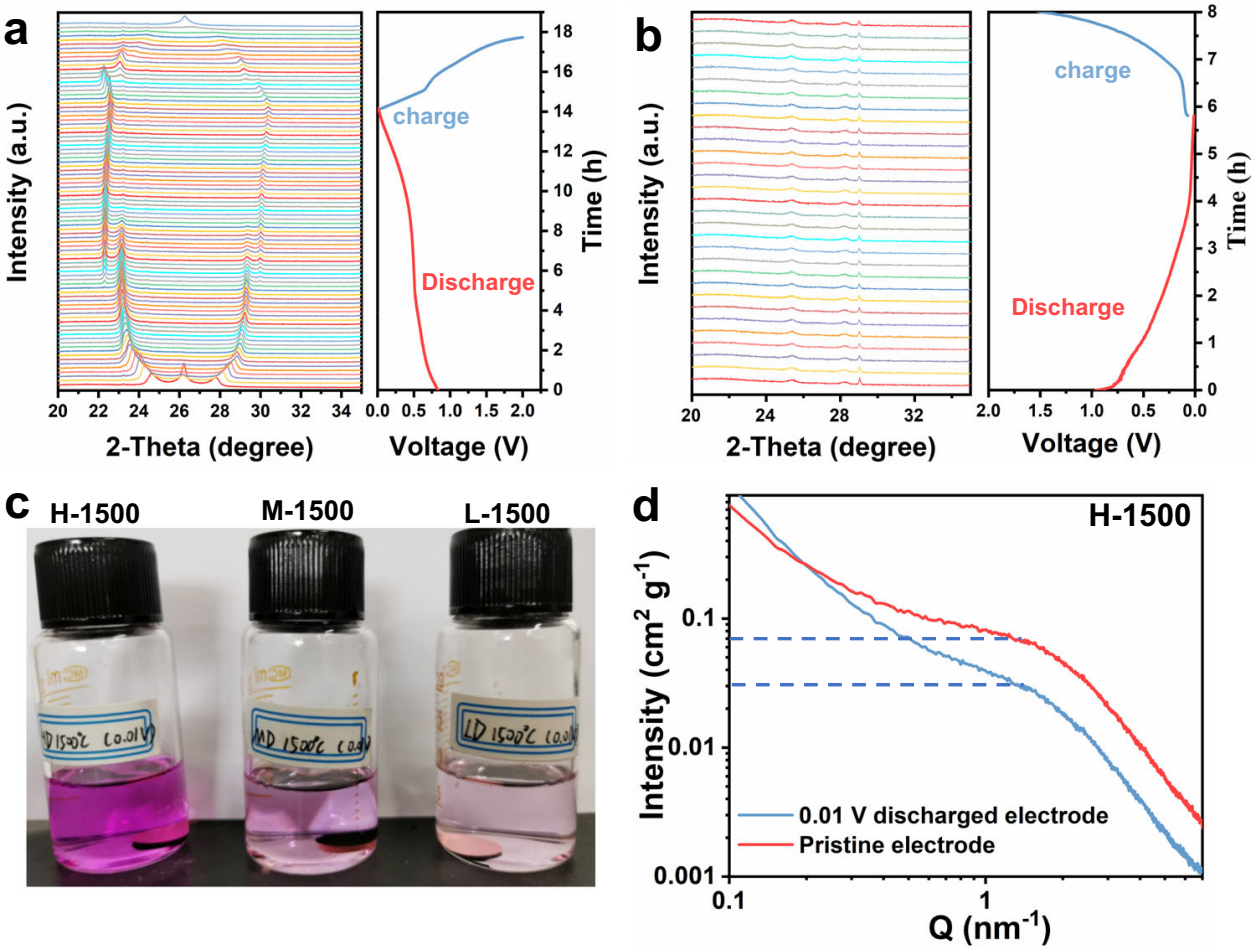
Analysis of sodium storage mechanism. In-situ XRD patterns of the graphite electrode (**a**) and the H-1500 electrode (**b**) during the first discharge-charge process at 100 mA g^−1^. **c** The optical photograph of hard carbon electrodes discharged to 0.01 V (vs. Na^+^/Na) soaked in the ethanol solution containing 1% phenolphthalein for 5 min (**d**) SAXS patterns of pristine and 0.01 V discharged H-1500 electrodes.

According to previously proposed adsorption/intercalation mechanism, the plateau capacity results from the accommodation of Na ions in curved graphene nanosheets of hard carbon, similar to Li ions insertion into graphite^10,33^. During this sodiation process, the interlayer spacing is inevitably expanded, which will lead to the obvious shift of diffraction peaks. As a reference, the process of Na^+^-ether insertion/extraction into/from graphite interlayer was firstly investigated via in-situ XRD technique. As seen, the diffraction peaks shift obviously (Fig. 7a). While for hard carbon electrode (H-1500), in addition to the characteristic peak of Be window at ~29 °^37^, the broad peak in a range of 24° and 26°, corresponding to the (002) graphitic plane of hard carbon remains the same, indicating that Na ions may not store in the interlayer spacing of hard carbon (Fig. 7b)^38^. Based on the adsorption-insertion-filling mechanism, it is probable that the quasi-metallic forms in closed pores at the plateau region^8^. To verify the presence of quasi-metallic sodium, Fig. 7c displays the optical photograph of hard carbon electrodes discharged to 0.01 V (vs. Na^+^/Na), which were soaked in the ethanol solution containing 1% phenolphthalein for 5 min^39^. It can be observed that the color of the ethanol-phenolphthalein solution deepens gradually with the increased plateau capacity of different derived-wood carbon, wherein H-1500 has more closed pores to store Na ions and the corresponding ethanol solution shows deeper red in contrast with L-1500 and M-1500. Meanwhile, SAXS tests of the pristine and discharged hard carbon electrodes were also employed^4,33^. As shown in Fig. 7d, when the electrode was discharged to 0.01 V, the SAXS intensity around Q = 1 nm^−1^ decreases significantly, which implies the decrease of closed pores because of the Na ions filling.

To verify the practical application potential of H-1500 electrode, the full-cell composed of the homemade Na_3_Fe_2_(PO_4_)P_2_O_7_@C (NFPP) cathode and H-1500 anode was constructed. Supplementary Fig. 16a is the charge/discharge curves of NFPP cathode at 100 mA g^−1^, and two plateaus at ~2.9 V and ~3.2 V can be observed, which are consistent with the previous literature^40^. NFPP delivers a discharge capacity of 86.6 mAh g^−1^ after 90 cycles at 100 mA g^−1^, corresponding to the capacity retention of ~99% (Supplementary Fig. 16b). The average Coulombic efficiency of NFPP is 99.5%, further demonstrating the stable cycling performance of this cathode material. NFPP also exhibits good rate performance with capacities of 87, 86, 84, 79 and 71 mAh g^−1^ at 0.2, 0.5, 1, 2 and 3 A g^−1^, respectively (Supplementary Fig. 16c, d). Supplementary Fig. 17a presents the charge/discharge curves of H-1500//NFPP full-cell at different current densities. H-1500//NFPP full-cell shows the initial reversible capacities of 346, 319 and 299 mAh g^−1^ (based on the mass of active anode material) at 0.1, 0.5 and 1 A g^−1^, respectively. H-1500//NFPP full-cell remains a discharge capacity of 254 mAh g^−1^ (based on the mass of active anode material) with a capacity retention of 79.6% after 100 cycles at 0.5 A g^−1^ (Supplementary Fig. 17b). Even at 1 A g^−1^, H-1500//NFPP full-cell still delivers a reversible capacity of 250 mAh g^−1^ (based on the mass of active anode material) with the capacity retention of 83.6%, demonstrating good cycling stability (Supplementary Fig. 17c, d).

## Discussion

In summary, the formation mechanism of closed pore, which is the main sodium storage structure in hard carbon, was established based on the wood-derived hard carbons. It was found that high crystalline cellulose content in nature wood could transform into long graphite-like layers to surround and shrink active sites to form closed pore structures. The existence of amorphous components (hemicellulose and lignin) not only helped to form nano-sized pores but also prevented the over-graphitization of carbon layer during the high-temperature carbonization. With the increase in carbonization temperature, the length of graphite-like carbon layer increased, which was beneficial to the formation of closed pore structure. Based on this carbonization model, the optimum H-1500 electrode exhibited a high reversible discharge capacity of 430 mAh g^−1^ at 20 mA g^−1^, good rate capability (175 mAh g^−1^ at 2000 mA g^−1^), and stable cycling performance (280 mAh g^−1^ after 400 cycles at 500 mA g^−1^). The assembled H-1500//NFPP full-cell remained a discharge capacity of 250 mAh g^−1^ (based on the mass of active anode material) with a capacity retention of 83.6% after 100 cycles at 1 A g^−1^, demonstrating good cycling stability. With the aid of in-situ XRD and SAXS, it was demonstrated that the plateau area mainly corresponded to the metallic Na cluster filling the closed pore. Therefore, H-1500 with abundant closed pores possessed more Na storage sites, contributing to its superior capacity. This work not only clarifies the closed pore formation mechanism for waste wood-derived carbon, but also offers a strategy to design high plateau-region hard carbon anodes for high-performance and low-cost SIBs in the future.

## Methods

### Synthesis of hard carbon samples

All precursors of hard carbon samples in this work were waste woods purchased on Taobao. These woods were divided into three categories according to their density and noted as L-wood (cork, 0.13 g cm^−3^), M-wood (yellow sandal, 0.392 g cm^−3^) and H-wood (rosewood, 0.891 g cm^−3^), respectively. The waste wood blocks were firstly cut into small pieces and then the precursors were directly carbonized for 2 h in a tube furnace under argon flow at 1100 °C, 1300 °C and 1500 °C, respectively. The heating rate was 2 °C min^−1^. Typically, these hard carbon samples derived from L-wood, which were prepared at different temperatures, were noted as L-1100, L-1300 and L-1500. The other samples were noted similarly. To investigate the influence of the disorder area on the formation of the closed pores, H-wood was pretreated via chemical methods in the following: firstly, H-wood blocks were crushed into powder. To remove the lignin in the precursor, a certain amount of H-wood powder was immersed into a yellow-green solution, in which 1 g NaClO_2_ (Macklin, 80%) and 2 mL CH_3_COOH (Macklin, AR) were dissolved in 150 mL deionized water^35^. The obtained mixture was stirred vigorously at 80 °C for different times (6, 12, 24 h), and then was separated via suction filtration. The treated powder was further washed with deionized water until the filtrate was almost neutral and dried at 80 °C overnight. To further remove the hemicellulose, the above acid-treated powder (24 h) was added in 200 mL NaOH (HUSHi, AR) aqueous solution with a gradient concentration of 6 wt%, 8 wt%, and 10 wt%, and kept at 80 °C for 12 h^34^. Finally, the alkali-treated powder was washed with deionized water until the pH of the filtrate was ~7 and dried at 80 °C overnight.

### Material characterization

Powder X-ray diffraction (XRD) was performed using a Rigaku diffractometer equipped with a Cu Kα radiation source (1.542 Å). Raman spectra were tested by the LabRAM HR800. Small-angle X-ray scattering (SAXS) data were collected with the X-ray diffractometer in a transmission and parallel-beam geometry with a Ni-filtered Cu Kα radiation and a scintillation point detector (Anton Paar SAXSess MC2). SAXS patterns of fully sodiated hard carbon samples were collected from the fabricated pouch cells with a metallic sodium reference and counter electrode, which were eventually discharged to 0.01 V (vs. Na^+^/Na) after 3 cycles at a specific current of 50 mA g^−1^. X-ray photoelectron spectroscopy (XPS, ESCALAB250Xi) was conducted to detect the surface valence state of samples. To avoid interference from the gas adsorbed in hard carbon, these powder samples were dried at 100 °C in a vacuum oven for 6h before the XPS test. ~2 mg hard carbon powder was distributed on the double side tape and fixed on the sample stage. Then the sample stage was transferred to the analysis room, which was vacuumed until the value reached to 10^−9^ Pa. A focused monochromatic Al Kα X-ray was used as an excitation source and high energy-resolution spectra collection were conducted with a spherical section analyzer. The hard carbon sample discharged to 0.01 V was collected from the cycled pouch cell, which was scraped from the Cu current collector and washed with dimethyl ether (DME) for drying in a vacuum before SAXS measurements. N_2_ and CO_2_ adsorption-desorption tests were conducted with BELSORP-mini II (MicrotracBEL Corp.) at 77K and 273K, respectively. Prior to the adsorption-desorption measurement, the samples were heat-treated at 300 °C for 5 h under vacuum to remove moisture trapped in the surface pores. The true densities of hard carbon samples were measured via helium gas pycnometry with the BELPycno density analyser (MicrotracBEL Corp.) and via n-butanol displacement pycnometry with a specific gravity bottle (Shibata Scientific Technology Ltd.) based on Japanese Industrial Standard (JIS) R7212:1995 as reported in the references^12^. Field-emission scanning electron microscopy (FE-SEM, Nova Nano SEM 230) and transmission electron microscopy (TEM, Titan G2 60-300) were utilized to characterize the morphological structures of samples.

### Electrochemical tests

CR2016 coin cells were assembled in an argon-filled glove box (Mikarouna) before testing the electrochemical performance of samples. Hard carbon anode was prepared by mixing 80 wt% hard carbon powder sample, 10 wt% acetylene black (Shenzhen Kejing) and 10 wt% polyvinylidene fluoride (PVDF, Shenzhen Kejing, HSV-900). The uniform slurry was prepared by adding the proper amount of N-methylpyrrolidone (Aladdin, AR) as solvent. And the slurry was cast on the copper foil and cut into 12 mm disks with a mass loading of around 1.8 mg cm^−2^ after the drying treatment at 100 °C for 12 h in a vacuum oven. The homemade Na circular pieces with a diameter of 12 mm that punched from the pressed Na foil, were used as the counter electrode and reference electrode. The glass fiber (Whatman) was used as the separator and the electrolyte was a solution of 1 M NaPF_6_ in dimethyl ether (Dodo Chem) in all cells. The hard carbon electrode in the pouch cell was prepared with a similar process to the electrode used in the coin cell except that the dried electrode was cut into a rectangle (width: 45 mm, height: 55 mm). The mass loading of hard carbon electrode in the pouch cell was ~80 mg. The homemade Na foils with a similar size to hard carbon anode were used and pressed on the Cu foil as the counter electrode and reference electrode. Ni tap needed to be welded on the Cu current collector. Eventually, hard carbon anode, glass fiber and Na foil were stacked in sequence and then encapsulated in aluminum-plastic film after adding a certain amount of electrolyte. For H-1500 electrode in the full-cell, the mass ratio of H-1500 powder, acetylene black and PVDF was 7:2:1, and the mass loading was ~1.5 mg cm^−2^. For NFPP electrode in the full-cell, the mass ratio of NFPP powder, acetylene black and PVDF was 8:1:1, and the mass loading of cathode disks with a diameter of 12 mm was 7–8 mg cm^−2^. According to the capacity of cathode and anode, the mass ratio between cathode and anode was ~4.58. Before assembling H-1500//NFPP full-cell, H-1500 electrode was precycled for 2 cycles at a specific current of 100 mA g^−1^ within the voltage range of 0.01–2 V in the half-cell. Both NFPP half-cell and H-1500//NFPP full-cell were cycled within the voltage range of 3.8-2 V. Galvanostatic charge/discharge tests were all conducted with Neware battery system in a thermotank at 30 °C.

### In-situ XRD measurement

The hard carbon/graphite (HUSHi, AR) electrode in the pouch cell was prepared with a similar process to the electrode used in the coin cell except that the prepared slurry was directly cast on the Be current collector with excellent X-ray penetration. This special electrode was dried in a vacuum oven at 100 °C for 12 h. The in-situ cell was fabricated in the glove box (Mikarouna). The homemade Na foils were used as the counter electrode and reference electrode. The glass fiber (Whatman) was used as the separator and the electrolyte was a solution of 1 M NaPF_6_ in dimethyl ether (Dodo Chem). The in-situ cell was cycled at a specific current of 100 mA g^−1^ between 0.01 and 2 V.

### Supplementary information

Supplementary Information

## Data Availability

The data that support the findings of this study are available within the article and its Supplementary Information files. All other relevant data supporting the findings of this study are available from the corresponding authors upon reasonable request.
