# Supplementary material for: Revealing the closed pore formation of waste wood-derived hard carbon for advanced sodium-ion battery
Source: Nat Commun. 2023 Sep 27;14:6024. doi: 10.1038/s41467-023-39637-5 (PMC10533848; doi:10.1038/s41467-023-39637-5)
Supplement: Supplementary file 1 — Supplementary Information [file 41467_2023_39637_MOESM1_ESM.pdf]

Supplementary Information

Revealing the closed pore formation of waste wood-derived hard carbon for advanced sodium-ion battery

Tang et al.

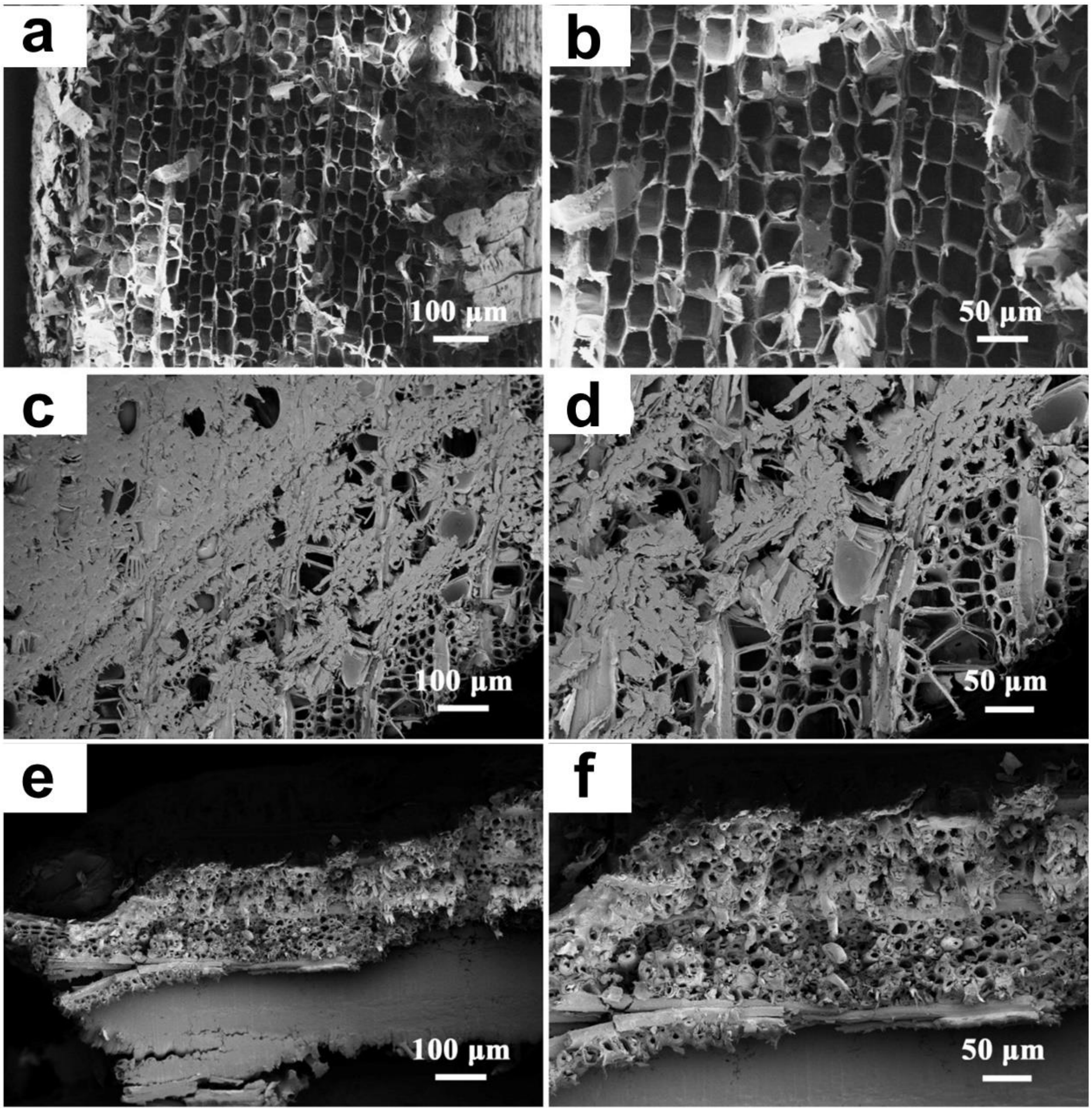

Supplementary Fig. 1 SEM images of different wood precursors. (**a**-**b**) L-wood, (**c**-**d**) M-wood, and (**e**-**f**) H-wood.

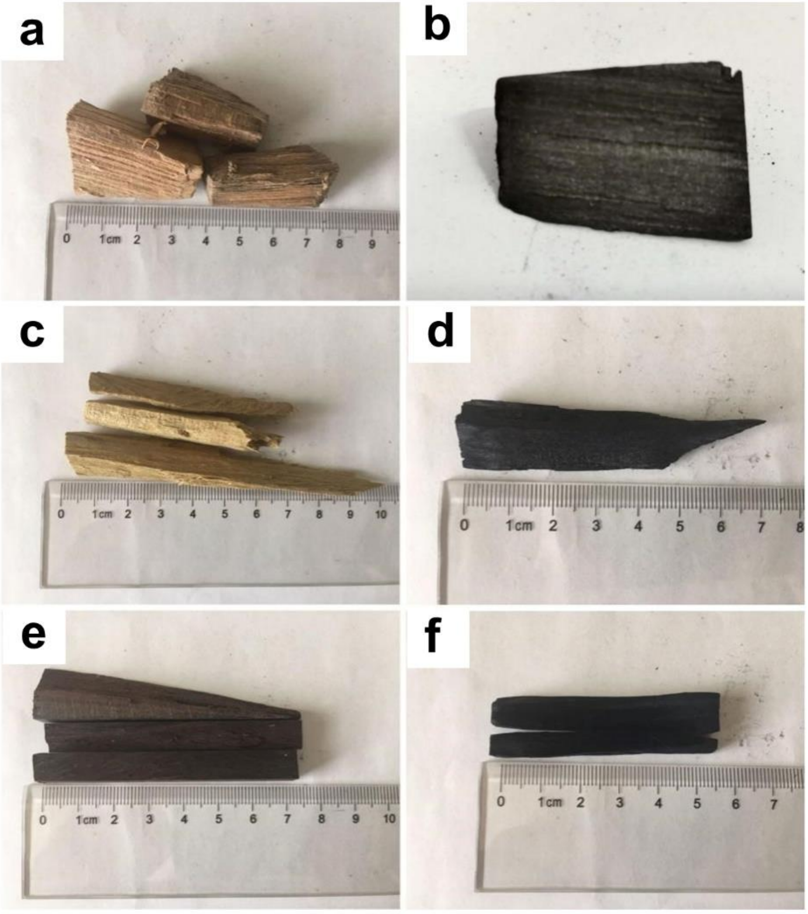

Supplementary Fig. 2 Optical photographs of different wood precursors and the corresponding carbonized samples. (**a**) L-wood, (**b**) L-1500, (**c**) M-wood, (**d**) M-1500, (**e**) H-wood and (**f**) H-1500.

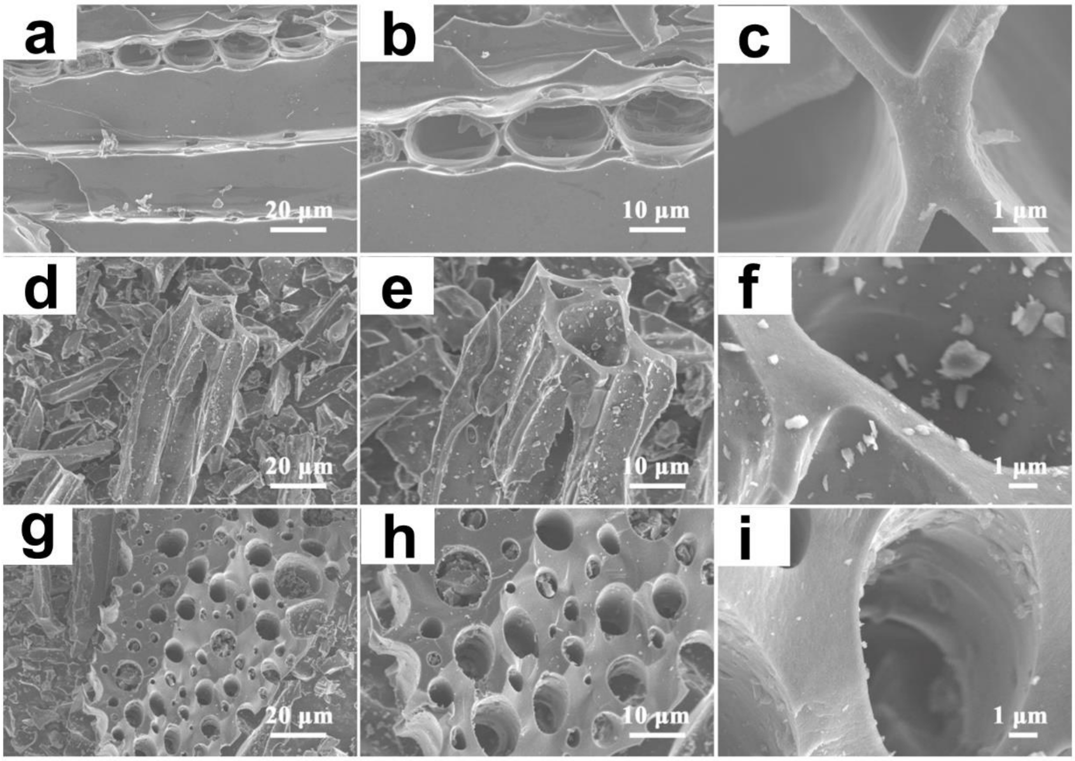

Supplementary Fig. 3 SEM images of different wood-derived hard carbon samples. (**a**-**c**) L-1500, (**d**-**f**) M-1500, and (**g**-**i**) H-1500.

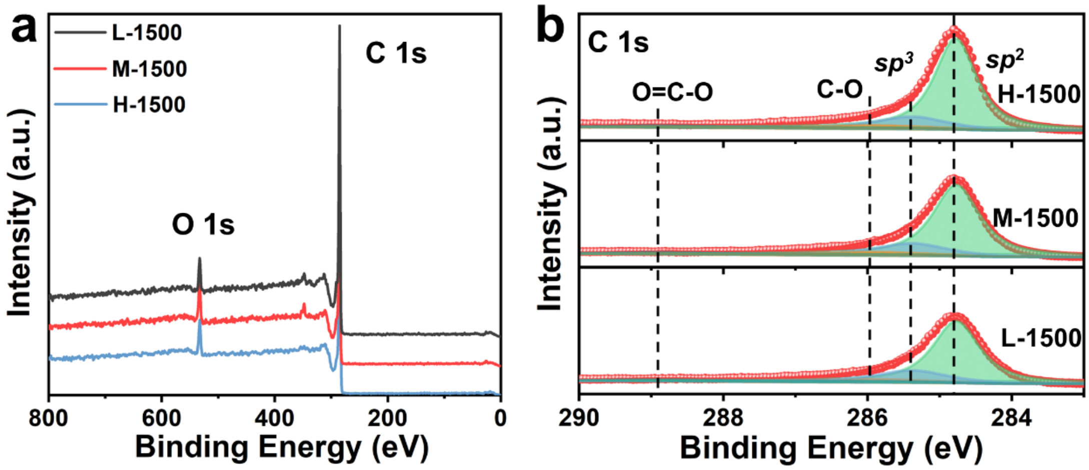

Supplementary Fig. 4 XPS spectra (**a**) and C 1s high-resolution spectra (**b**) of hard carbon samples derived from wood precursors with different crystalline cellulose content.

Supplementary Table 1 The element contents of C and O in L-1500, M-1500 and H-1500 samples

Samples	Atomic %	
	C	O
L-1500	94.38	5.62
M-1500	94.72	5.28
H-1500	95.19	4.81

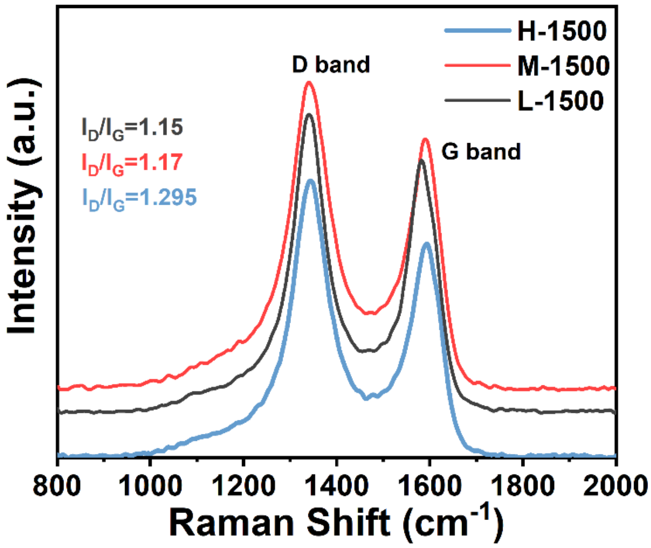

Supplementary Fig. 5 Raman spectra of hard carbon samples derived from wood precursors with different crystalline cellulose content.

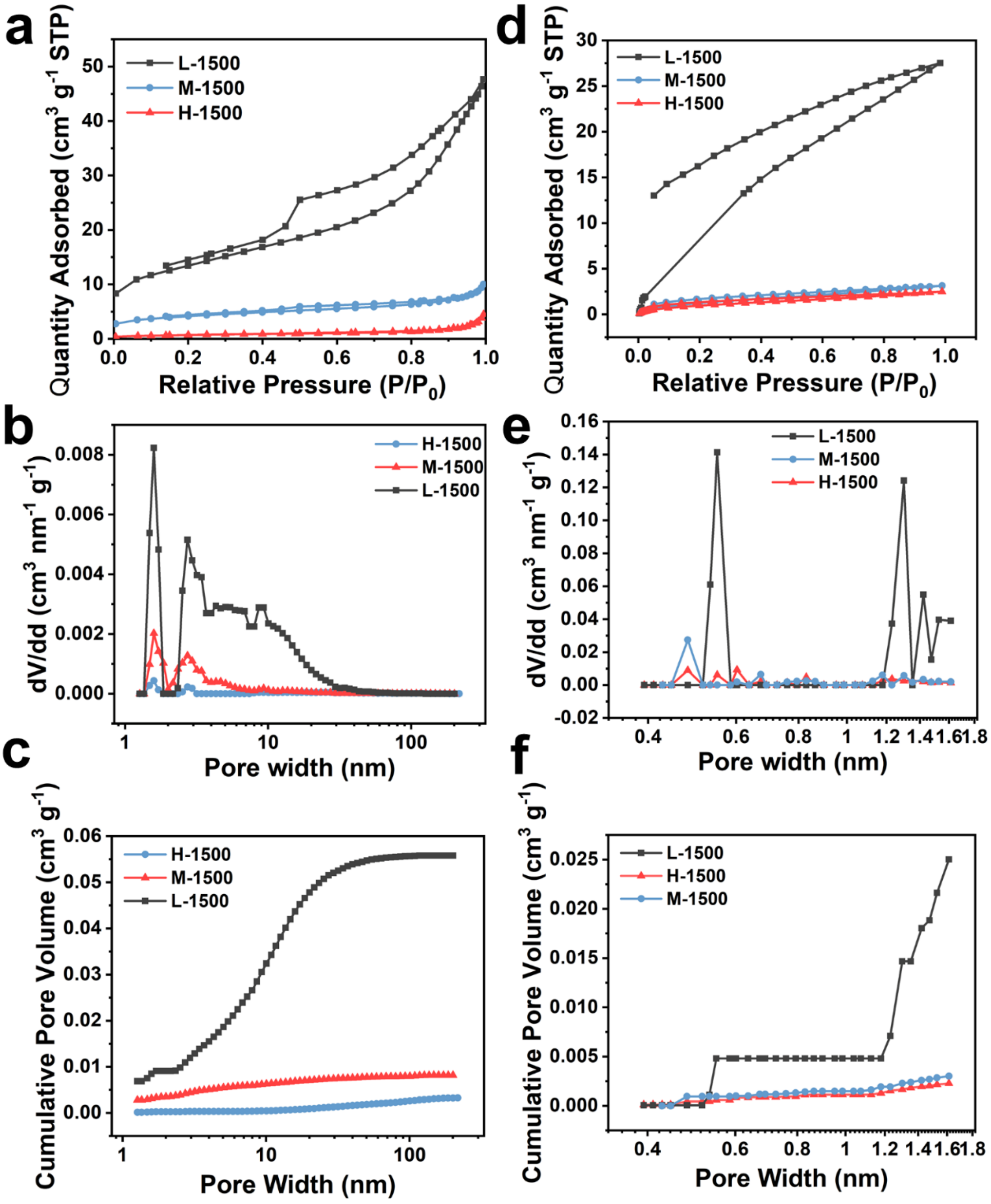

Supplementary Fig. 6 N_2 physisorption test: (**a**) N_2 adsorption−desorption isotherms, (**b**) differential pore size distribution and (**c**) cumulative pore volume as calculated from DFT. CO_2 physisorption test: (**d**) CO_2 adsorption−desorption isotherms, (**e**) differential pore size distribution and (**f**) cumulative pore volume as calculated from non-local density functional theory (NLDFT).

According to N_2 adsorption−desorption isotherms and BET model in **Supplementary Fig. 6a**, the specific surface areas (SSAs) of L-1500, M-1500 and H-1500 are 47.6, 14.6 and 2.6 m^2 g^{-1}, respectively. The differential pore size distribution

curves and the cumulative pore volume further prove the presence of rich micropores and mesopores in L-1500, and H-1500 possesses the lowest content of micropores and mesopores (**Supplementary Fig. 6b**, **c**). CO_2 adsorption/desorption measurements were further applied to investigate ultramicropores in hard carbon samples. According to CO_2 adsorption−desorption isotherms and BET model in **Supplementary Fig. 6d**, the SSAs of L-1500, M-1500 and H-1500 are 12.6, 5.3 and 4.1 m^2 g^{-1}, respectively. Pore size distribution curves indicate that the ultramicropores in L-1500 are located around 0.56 nm, while the ultramicropores in M-1500 and H-1500 are located around 0.48 nm (**Supplementary Fig. 6e**). Moreover, the pore volume values of M-1500 and H-1500 are also much less than that of L-1500 (**Supplementary Fig. 6f** and **Supplementary Table 2)**.

Supplementary Table 2 Pore characteristics of the materials determined from N_2 and CO_2 physisorption

Samples	$SSA_{BET,N2}$/m^2 g^{-1}	$SSA_{BET,CO2}$/m^2 g^{-1}	$V_{N2,<200\ nm}$/cm^3 g^{-1}	$V_{CO2,<1.6\ nm}$/cm^3 g^{-1}
L-1500	47.6	12.6	0.05579	0.025
M-1500	14.6	5.3	0.00816	0.003
H-1500	2.6	4.1	0.00326	0.0023

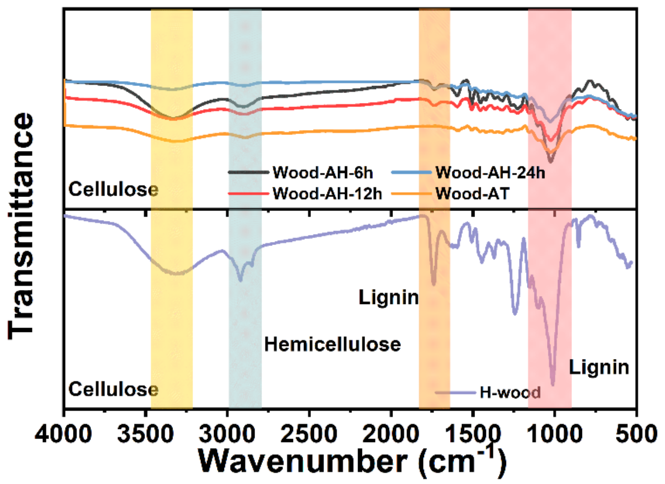

Supplementary Fig. 7 FTIR spectra of H-wood and wood precursors pretreated with acid (Wood-AH-6h, Wood-AH-12h and Wood-AH-24h) or alkali (Wood-AT).

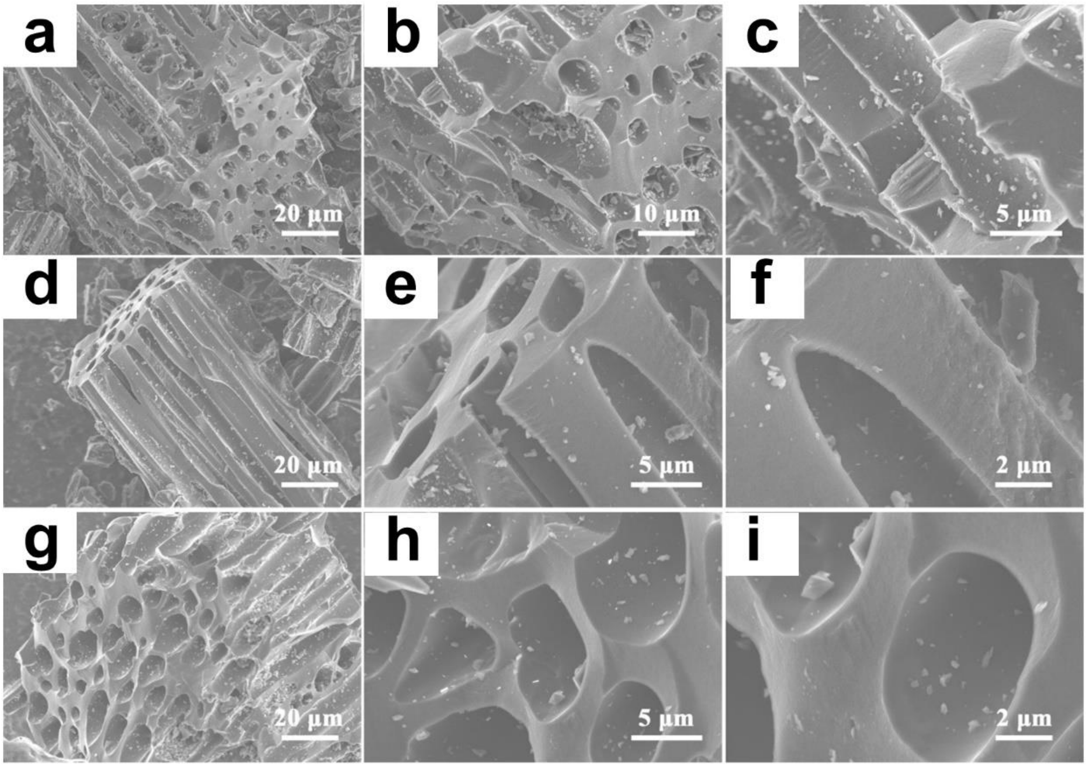

Supplementary Fig. 8 SEM images of (**a**-**c**) H-1100, (**d**-**f**) H-1300, and (**g**-**i**) H-1500.

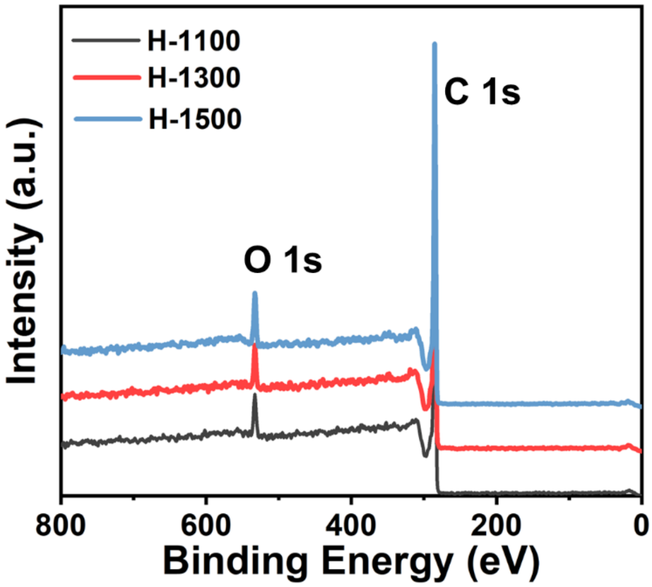

Supplementary Fig. 9 XPS spectra of H-1100, H-1300 and H-1500 samples.

Supplementary Table 3 The element contents of C and O in H-1100, H-1300 and H-1500 samples

Samples	Atomic %	
	C	O
H-1100	92.81	7.19
H-1300	92.14	7.86
H-1500	95.19	4.81

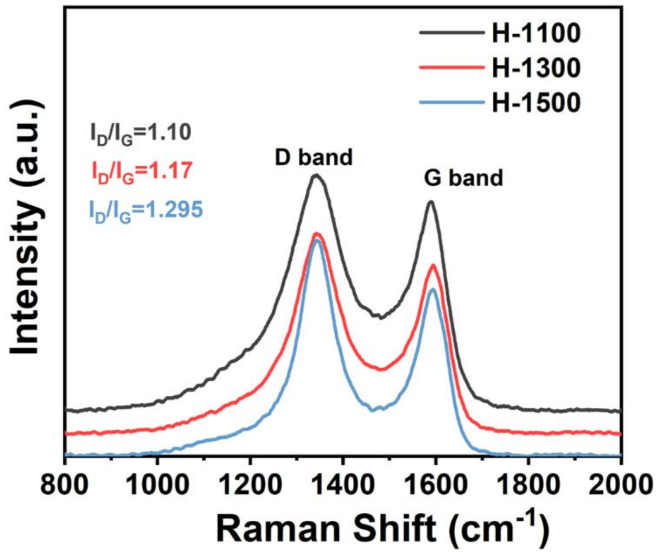

Supplementary Fig. 10 Raman spectra of H-1100, H-1300 and H-1500 samples.

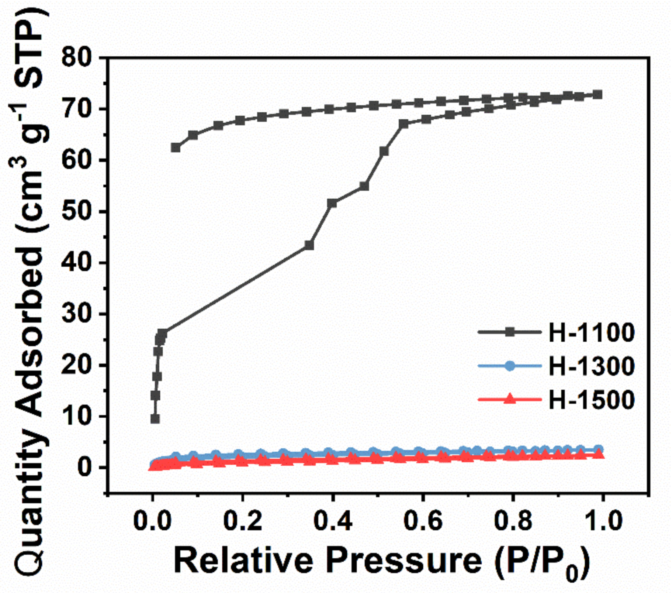

Supplementary Fig. 11 CO_2 adsorption−desorption isotherms of H-1100, H-1300 and H-1500.

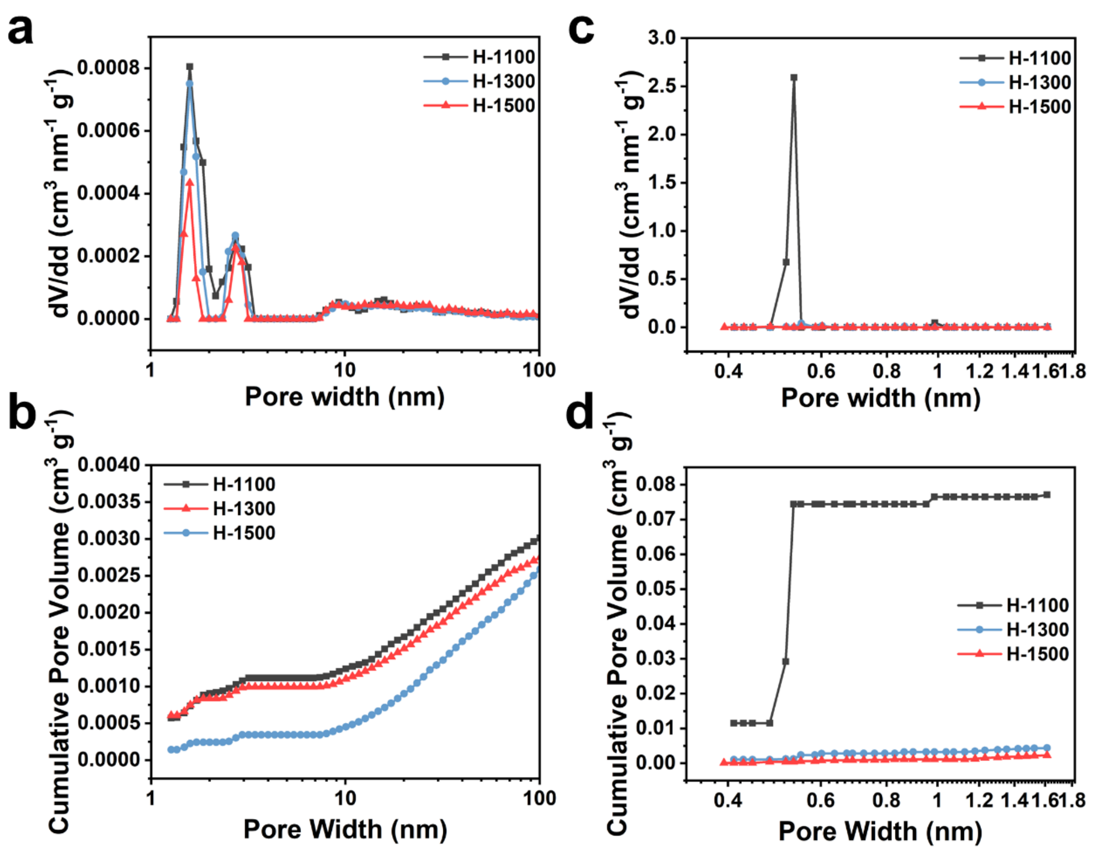

Supplementary Fig. 12 N_2 physisorption test: (**a**) differential pore size distribution and (**b**) cumulative pore volume as calculated from DFT. CO_2 physisorption test: (**c**) differential pore size distribution and (**d**) cumulative pore volume as calculated from NLDFT.

Supplementary Table 4 Pore characteristics of the H-wood derived hard carbon materials determined from N_2 and CO_2 physisorption

Samples	$SSA_{BET,N2}$/m^2 g^{-1}	$SSA_{BET,CO2}$/m^2 g^{-1}	$V_{N2,<234\ nm}$/cm^3 g^{-1}	$V_{CO2,<1.6\ nm}$/cm^3 g^{-1}
H-1100	4.2	134.0	0.0035	0.077
H-1300	4.5	8.0	0.0031	0.0044
H-1500	2.6	4.1	0.00326	0.0023

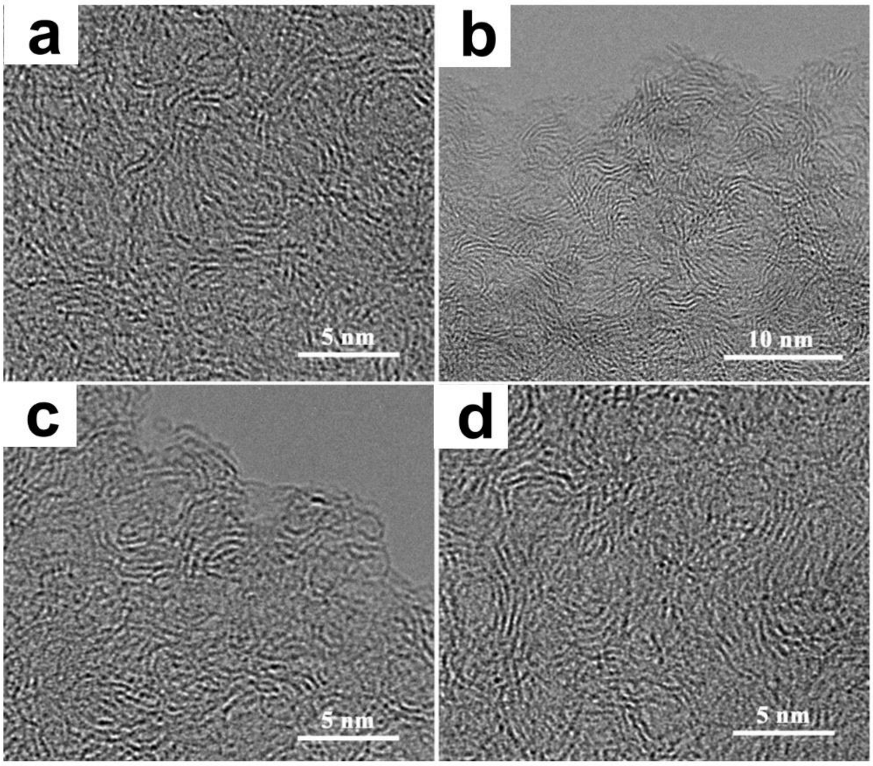

Supplementary Fig. S13 TEM images of H-wood-derived carbon samples synthesized at different temperatures. (**a**) 1100 ºC, (**b**-**c**) 1300 ºC, and (**d**) 1500 ºC.

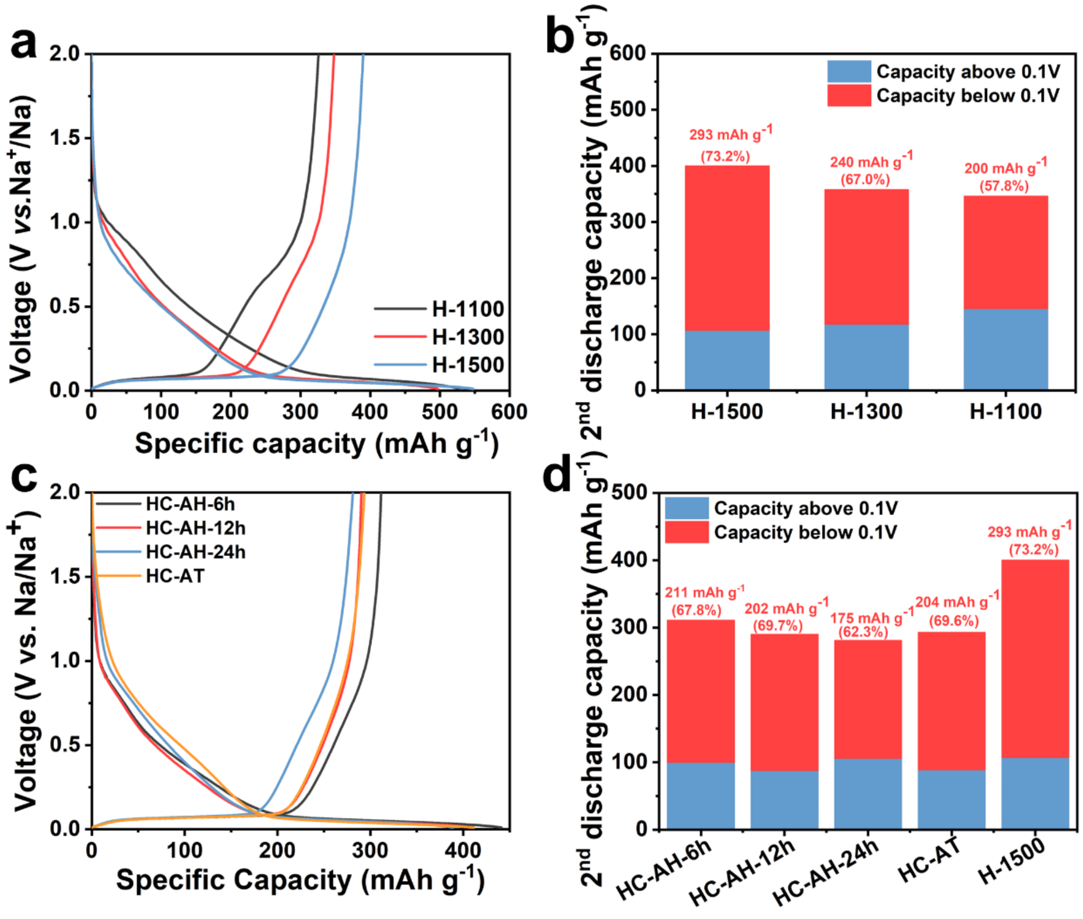

Supplementary Fig. 14 Electrochemical performance comparison. (**a**) The initial discharge-charge profiles of H-1100, H-1300 and H-1500 samples at 50 mA g^{-1}. (**b**) The second discharge capacity of samples contributed from slope and plateau region for H-1100, H-1300 and H-1500. (**c**) The first discharge-charge profiles for HC-AH-6h, HC-AH-12h, HC-AH-24h, and HC-AT at a current rate 50 mA g^{-1} in Na half-cell. (**d**) The second discharge capacity contributed from slope and plateau region for HC-AH-6h, HC-AH-12h, HC-AH-24h, HC-AT and H-1500.

The carbonization temperatures also play great effect on the capacity of hard carbon. **Supplementary Fig. 14a** shows the charge-discharge curves in the initial three cycles of H-1100, H-1300, and H-1500 at a specific current of 50 mA g^{-1}. H-1500 demonstrates the highest capacity of 400 mAh g^{-1}, higher than those of H-1100 (320 mAh g^{-1}) and H-1300 (342 mAh g^{-1}). **Supplementary Fig. 14b** shows the capacity contributed from the plateau region and slope region. As the pyrolysis temperature increases, the ratio of plateau capacity is enhanced.

The effect of closed pore structure on capacity is further confirmed by the electrochemical performance comparison among H-1500, HC-AH-6h, HC-AH-12h, HC-AH-24h and HC-AT. The pretreatment can remove the amorphous composition in wood precursor and eventually facilitates the formation of closed pore. As displayed in **Supplementary Fig. 14c**, the initial reversible capacity of hard carbon decreases with the prolonged treating time. H-1500 demonstrates the highest capacity due to its high closed pore content among all hard carbon samples. **Supplementary Fig. 14d** further illustrates that the plateau capacities of H-1500, HC-AH-6h, HC-AH-12h, HC-AH-24h and HC-AT are 293, 211, 202, 175, 204 mAh g^{-1}, respectively. Based on the above discussion, it is sure that the closed pore structure in hard carbon plays a key role in the plateau capacity contribution.

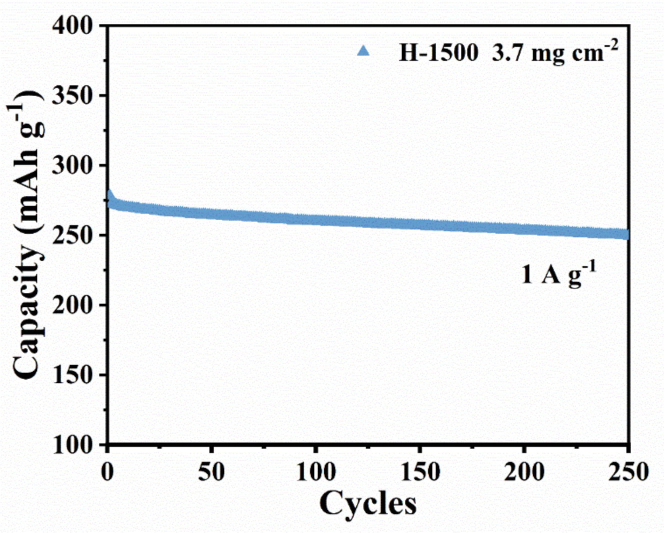

Supplementary Fig. 15 Cycling performance of H-1500 electrode with a high mass loading of 3.7 mg cm^{-2} at 1 A g^{-1}.

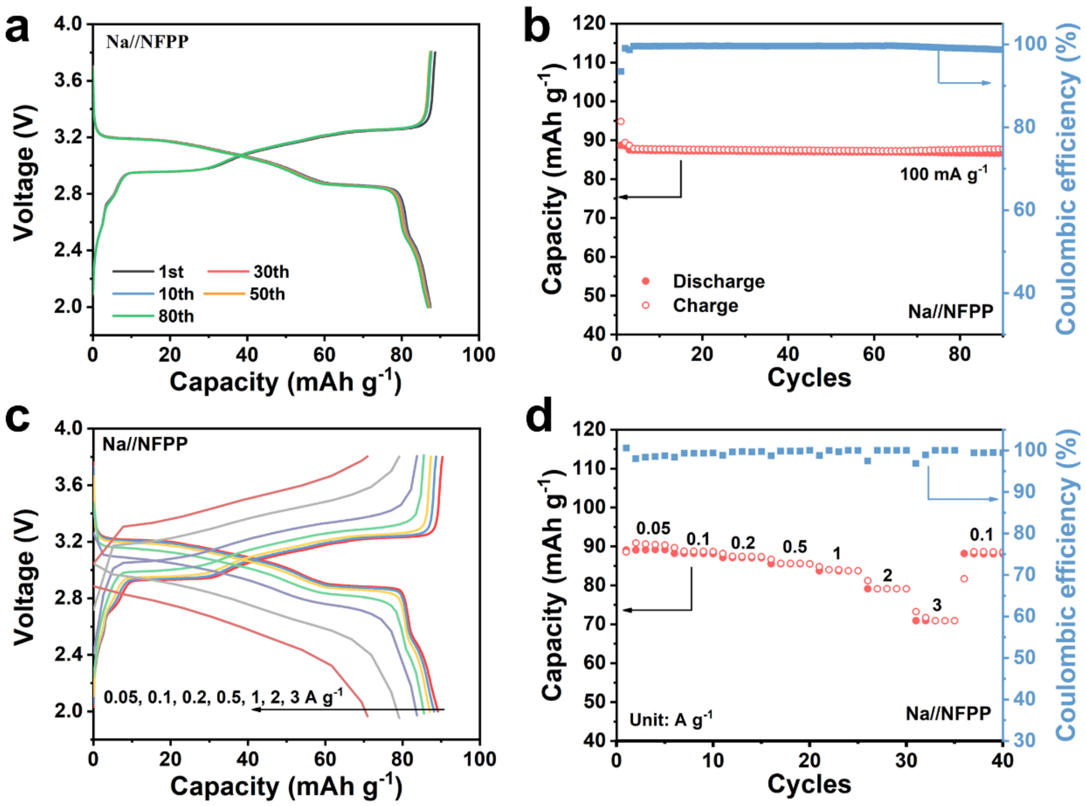

Supplementary Fig. 16 Electrochemical performance of NFPP cathode. (**a**) Charge/discharge curves at different cycles and (**b**) cycling performance of NFPP cathode at 100 mA g^{-1}. (**c**) Charge/discharge curves at different current densities and (**d**) rate performance of NFPP cathode.

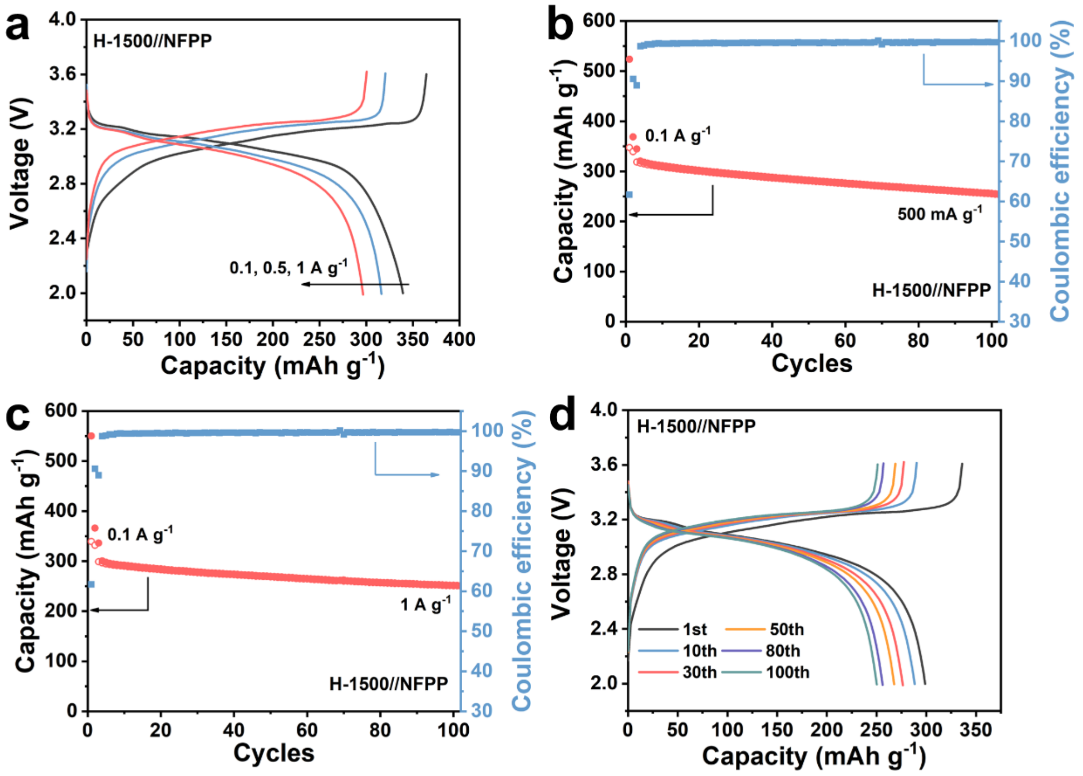

Supplementary Fig. 17 Electrochemical performance of full-cell. (**a**) Charge/discharge curves at different current densities. Cycling performance of H-1500//NFPP full-cell at (**b**) 0.5 A g^{-1} and (**c**) 1 A g^{-1}. (**d**) Charge/discharge curves of H-1500//NFPP full-cell at different cycles when the current density is 1 A g^{-1}.
